# Role of Recipient Susceptibility Factors During Contact-Dependent Interbacterial Competition

**DOI:** 10.3389/fmicb.2020.603652

**Published:** 2020-11-12

**Authors:** Hsiao-Han Lin, Alain Filloux, Erh-Min Lai

**Affiliations:** ^1^Institute of Plant and Microbial Biology, Academia Sinica, Taipei, Taiwan; ^2^MRC Centre for Molecular Bacteriology and Infection, Department of Life Sciences, Imperial College London, London, United Kingdom

**Keywords:** recipient susceptibility factor, antibacterial activity, bacterial secretion system, CDI, T6SS, effector, Cdz

## Abstract

Bacteria evolved multiple strategies to survive and develop optimal fitness in their ecological niche. They deployed protein secretion systems for robust and efficient delivery of antibacterial toxins into their target cells, therefore inhibiting their growth or killing them. To maximize antagonism, recipient factors on target cells can be recognized or hijacked to enhance the entry or toxicity of these toxins. To date, knowledge regarding recipient susceptibility (RS) factors and their mode of action is mostly originating from studies on the type Vb secretion system that is also known as the contact-dependent inhibition (CDI) system. Yet, recent studies on the type VI secretion system (T6SS), and the CDI by glycine-zipper protein (Cdz) system, also reported the emerging roles of RS factors in interbacterial competition. Here, we review these RS factors and their mechanistic impact in increasing susceptibility of recipient cells in response to CDI, T6SS, and Cdz. Past and future strategies for identifying novel RS factors are also discussed, which will help in understanding the interplay between attacker and prey upon secretion system-dependent competition. Understanding these mechanisms would also provide insights for developing novel antibacterial strategies to antagonize aggressive bacteria-killing pathogens.

## Introduction

Bacteria are one of the most abundant forms of life on earth, and they have developed multiple strategies to compete with each other and fight for limited resources and space (Foster and Bell, 2012; Ghoul and Mitri, 2016). An effective strategy in this war game is to deliver toxins into opponents in order to kill them or challenge their fitness (Costa et al., 2015; Filloux and Sagfors, 2015; Green and Mecsas, 2016; Coulthurst, 2019; Klein et al., 2020). These toxins are deadly when they destroy the cell membrane integrity (e.g., peptidoglycan hydrolase, amidase, lipase, or pore-forming protein) or degrade nucleic acid (DNase, RNase, or tRNase) (Willett et al., 2015b; Lien and Lai, 2017). The challenge is to deliver efficiently one or more toxins to the appropriate destination. Thus, sophisticated mechanisms are evolved to allow the toxins to transport across the membranes and outreaching their molecular targets of the recipient cells while avoiding self-intoxication or intoxication of kins. For the latter, it is most remarkable that each toxin is encoded together with a specific immunity protein that would prevent toxicity, usually through direct protein–protein interaction.

**FIGURE 1:**
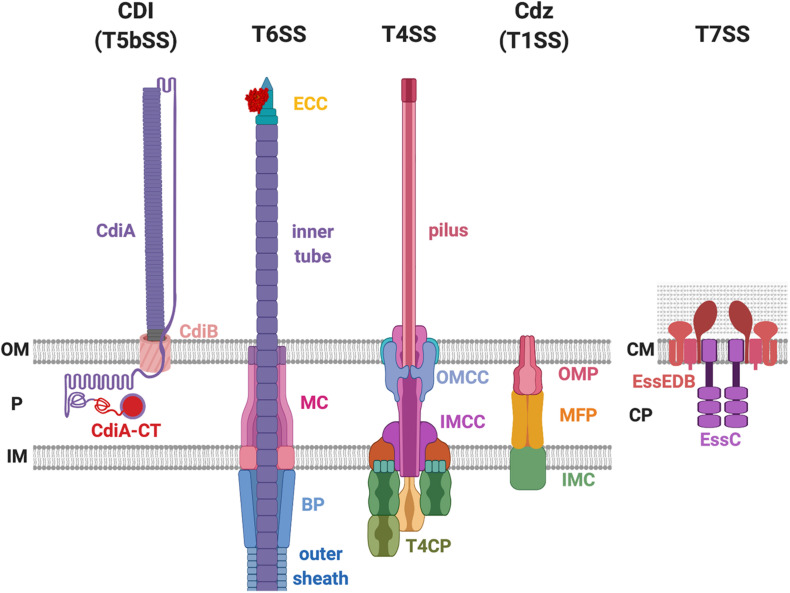
Bacterial secretion systems proven to deliver antibacterial toxins. The contact-dependent inhibition (CDI) system belongs to the type Vb secretion system (T5bSS), which is composed of outer membrane (OM) barrel CdiB and the surface-exposing CdiA protein. The type VI secretion system (T6SS) consists of the membrane complex (MC), baseplate (BP) complex, effector-containing complex (ECC), the outer sheath, and the inner tube. The type IV secretion system (T4SS) is composed of the outer membrane (OM) core complex (OMCC), inner membrane (IM) core complex (IMCC), type IV coupling protein (T4CP), and the pilus. The CDI by glycine-zipper protein (Cdz) belongs to the T1SS that consists of three proteins: the OM protein (OMP), the membrane fusion protein (MFP), and the IM component (IMC). The T7SS exists in the Gram-positive bacteria that only have one lipid bilayer. The T7SS is composed of the EssE, EssD, EssB, and the EssC protein. P, periplasm; CM, cell membrane; CP, cytoplasm.

There are several protein secretion systems that have been designed by bacteria for robust and efficient delivery of protein from the cytosol across the cell envelope. Among the nine identified so far (reviewed in Filloux and Sagfors, 2015; Christie, 2019), some have a proven capability to deliver antibacterial toxins (Aoki et al., 2005; Hood et al., 2010; Souza et al., 2015; Cao et al., 2016; García-Bayona et al., 2017). These are the type I secretion system (T1SS), type IV secretion system (T4SS), type V secretion system (T5SS) and here more specially those called contact-dependent inhibition (CDI) system, type VI secretion system (T6SS), and type VII secretion system (T7SS) (Figure 1). There are also a number of other examples such as colicins whose delivery does not involve the assembly of a supramolecular secretion machine but relies upon cell lysis (Cascales et al., 2007).

To date, several papers have provided thorough overviews of the molecular mechanisms associated with these secretion systems, such as structural organization, regulatory networks, or the identity and mode of action of a repertoire of antibacterial toxins (Costa et al., 2015; Filloux and Sagfors, 2015; Green and Mecsas, 2016; Coulthurst, 2019; Klein et al., 2020). It is seldom considered what in the recipient cells might be required for an attack to be successful such as recipient susceptibility (RS) factors. To date, the best characterized RS factors are the ones recognized by the CDI system (Ruhe et al., 2020) and have been mostly identified by genetic screens (Aoki et al., 2008; Ruhe et al., 2014, 2017; Willett et al., 2015a; Jones et al., 2017). Recently, few other RS factors were identified in association with the T6SS or a novel CDI by glycine-zipper protein (Cdz) system, notably by screening resistant mutants or using knowledge-based approaches (Whitney et al., 2015; Mariano et al., 2018; García-Bayona et al., 2019; Lin et al., 2020). Our current knowledge suggests that CDI employs a receptor-based recognition mechanism for toxin delivery between close siblings at intraspecies levels, while T6SS uses mechanical force for toxin delivery into a wide range of recipient cells in a receptor-independent manner. The present review will focus on the CDI, T6SS, and Cdz by describing the secretion machine and their toxins with further highlights on the specific RS factors (e.g., membrane receptors and cytoplasmic proteins) that maximize delivery and activity of incoming toxins. In addition, we also discussed the current and potential strategies for identifying novel RS factors and proposed RS-mediated antibacterial strategies. The knowledge learned from these three systems may provide new insights to identify and investigate RS factors involved in regulating antibacterial activity from other systems, notably T4SS and T7SS.

## Contact-Dependent Growth Inhibition Systems

### The Discovery, the Players, and the Mode of Action

Aoki et al. (2005) reported that wild-type *Escherichia coli* strain EC93 inhibits the growth of the laboratory strain MG1655 in a one-inhibits-many manner requiring direct cell-to-cell contact. Therefore, the authors defined this phenomenon as CDI. It was later on discovered that the CDI system is widely distributed in the α-, β-, and γ-proteobacteria (Aoki et al., 2010; Poole et al., 2011) and is functional in many species like *E. coli*, *Burkholderia pseudomallei*, *Dickeya dadantii*, *Pseudomonas aeruginosa*, and *Acinetobacter baylyi* (Aoki et al., 2010; Kiel et al., 2012; De Gregorio et al., 2018; Allen and Hauser, 2019).

The genes responsible for CDI in *E. coli* are *cdiB*, *cdiA*, and *cdiI.* The *cdiI* gene encodes an immunity protein that protects the attacker cell from self-intoxication (Aoki et al., 2005, 2010). The toxin domain is located at the C-terminal end of CdiA (termed CdiA-CT), which otherwise is a large protein (∼180–640 kDa) that forms a long filamentous structure with its N-terminus attached on the cell surface (Figure 1; Aoki et al., 2010; Willett et al., 2015b). CdiB is an outer-membrane beta-barrel protein that allows translocation and presentation of the CdiA toxin at the cell surface of the attacker cell (Figure 1). Both CdiB and CdiA are required to successfully inhibit the growth of the recipient cells (Aoki et al., 2005). CdiB and CdiA belong to a two-partner secretion (TPS) system also known as T5bSS, a subtype of the T5SS (Aoki et al., 2005; Filloux and Sagfors, 2015).

**FIGURE 2:**
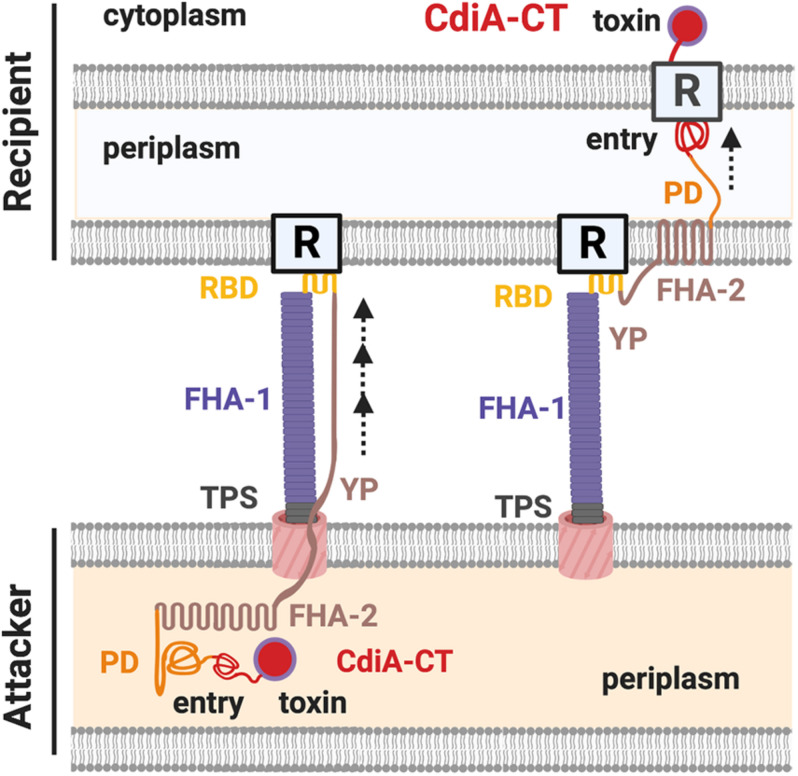
The CdiA domains and the contact-dependent inhibition (CDI) working model. The domains of a CdiA from the N-terminus are the conserved two-partner secretion (TPS) transport domain, the filamentous hemagglutinin domain 1 (FHA-1), the receptor-binding domain (RBD), the Tyr/Pro-enriched (YP) domain, the second FHA domain (FHA-2), the pre-toxin (PD) domain, and the C-terminus toxin domain (CdiA-CT). The CdiA-CT is further divided into the N-terminus entry subdomain and the C-terminus toxin domain. In the resting state, the FHA-2, PD, and the CdiA-CT remain in the attacker cell, while the TPS, FHA-1, RBD, and the YP domains are exposed in the extracellular milieu of the cell surface. Upon recognizing the outer membrane (OM) receptor (R) of a recipient cell by the RBD domain of CdiA, FHA-2 exposes and assembles into the recipient OM and translocates the CdiA-CT into the recipient periplasm. The entry domain then recognizes the IM receptor and translocates the toxin domain into the cytoplasm, where the toxin exerts its toxicity.

The domains of CdiA toxin include the N-terminal Sec-dependent signal peptide, the conserved TPS transport domain, the filamentous hemagglutinin adhesin domain 1 (FHA-1), the receptor binding domain (RBD), the Tyr/Pro-enriched (YP) domain, the second FHA domain (FHA-2), the pre-toxin domain (PD), and the C-terminal toxin domain (CdiA-CT) (Figure 2; Willett et al., 2015a; Ruhe et al., 2017, 2018). Both electron cryotomography and biochemical data support that the structure of CdiA resembles a U-shape hair clip and the RBD domain is at the bending point (Ruhe et al., 2018). One leg of the CdiA hair clip is composed of an elongated FHA-1 beta-helix whose filamentous structure extends out from the cell surface and another leg of CdiA is likely composed of the YP domain, which is required for cell surface presentation (Ruhe et al., 2017, 2018). The FHA-2 domain is required for toxin delivery into the recipient cell (Ruhe et al., 2018). The function of the PD domain is unclear, but it contains a VENN motif, which is highly conserved among different CdiA-harboring species and precisely precedes the N-terminal region of the toxin domain CdiA-CT (Aoki et al., 2010; Ruhe et al., 2018). The CdiA-CT consists of the N-terminal entry domain and the C-terminal toxin domain (Figure 2; Ruhe et al., 2018). The N-terminal entry domain is responsible for interacting with recipient’s inner membrane (IM) factor(s), and such interaction controls CdiA-CT toxin translocation into the recipient cytosol. Of note, the FHA-2 and the CdiA-CT reside in the attacker periplasm, and the delivery resumes only after the RBD domain binds to its specific recipient receptor. The FHA-2 domain is tightly associated with the recipient-cell outer membrane (OM), and this interaction is required for CdiA-CT translocation into the periplasm of target bacteria. The structure of FHA-2 is unknown but predicted to resemble an LptD lipopolysaccharide transporter consisting of a 26-stranded beta-barrel in the OM. These findings led to a proposed model that the FHA-2 domain may assemble into a transmembrane conduit for toxin translocation into the periplasm of the recipient cell (Figure 2; Ruhe et al., 2018).

### CdiA Toxins Recognize Specific Outer Membrane Receptors of Recipient Cells

#### BamA Is the Outer Membrane Receptor Recognized by CDI^EC93^

A contact-dependent process combined with the presence of CdiA at the cell surface of the attacker raised a question as to whether a cell surface receptor in the recipient cell is involved in docking/recognition of CdiA. If this was the case, then a variant of recipient cells for which the receptor is lacking or altered would become CDI-resistant (CDI^R^). Using CDI^EC93^ as a model, a transposon (Tn)-based mutagenesis screening led to the identification of such CDI^R^ mutants that have Tn insertion in either *acrB* or the promoter region of *bamA* (Table 1; Aoki et al., 2008).

**TABLE 1:** Recipient susceptibility (RS) factors required for bacterial secretion system toxins.

RS factor	Known or putative function of RS	Toxin	Secretion system	Toxin function
**OM factors**				
BamA	Translocator	CdiA^EC93^	CDI	Putative pore-forming
OmpC/OmpF	Translocator	CdiA^EC536^	CDI	tRNase
	Translocator	CdiA^ECL^	CDI	16S rRNase
Tsx, OmpT	Translocator	CdiA^STEC031^	CDI	tRNase
PerA	Translocator	CdzC/Cdzd	Cdz	unknown
**Periplasmic factors**				
DsbA	Activator	Ssp2	T6SS	Peptidoglycan hydrolases
	Activator	Ssp4	T6SS	unknown
**IM factors**				
AcrB	Translocator/activator?	CdiA^EC93^	CDI	Putative pore-forming
FtsH	Translocator	CdiA^EC536^	CDI	tRNase
		Cdi^AECL^	CDI	16S rRNase
PstG	Translocator	CdiA^STECO31^	CDI	tRNase
	Translocator	CdiA^NC101^	CDI	tRNase
	Translocator	CdiA^EC3006^	CDI	tRNase
YciB	Translocator	CdiA^EC869^	CDI	tRNase
MetI	Translocator	CdiA^MH813^	CDI	Nuclease
GltK	Translocator	CdiA^TT01^	CDI	Nuclease
RbsC	Translocator	CdiA^Dd3937^	CDI	DNase
**Cytosol factors**				
CysK	Activator	CdiA^EC536^	CDI	tRNase
EF-Tu/EF-Ts	Activator	CdiA^NC101^	CDI	tRNase
	Activator	CdiA^EC3006^	CDI	tRNase
	Activator	CdiA^EC869^	CDI	tRNase
	unknown	Tse6	T6SS	NAD(P) + Glycohydrolase

*CDI, contact-dependent inhibition; OM, outer membrane; T6SS, type VI secretion system; IM, inner membrane; Cdz, CDI by glycine zipper protein.*

BamA is an OM protein at the core of the beta-barrel assembly machinery (BAM) complex and required for proper assembly/insertion of other beta-barrel proteins in the OM (Rigel and Silhavy, 2012). As *bamA* is an essential gene, the *bamA*-mutated CDI^R^ mutant is not a null mutant but a knockdown mutant with five-fold less expression (Aoki et al., 2008). The biogenesis-inactive version of BamA, as well as other BAM complex variants, remains capable to mediate CDI, indicating that the presence of BamA but not the function of the BAM complex is required for CDI (Aoki et al., 2008). Treatment of bacterial cultures using anti-BamA antibody that recognizes the recipient BamA on the cell surface disrupted the attacker-recipient cell recognition and thus the CDI-mediated growth inhibition (Aoki et al., 2008). The results strongly support the idea that BamA is an OM receptor of the CdiA^EC93^.

**FIGURE 3:**
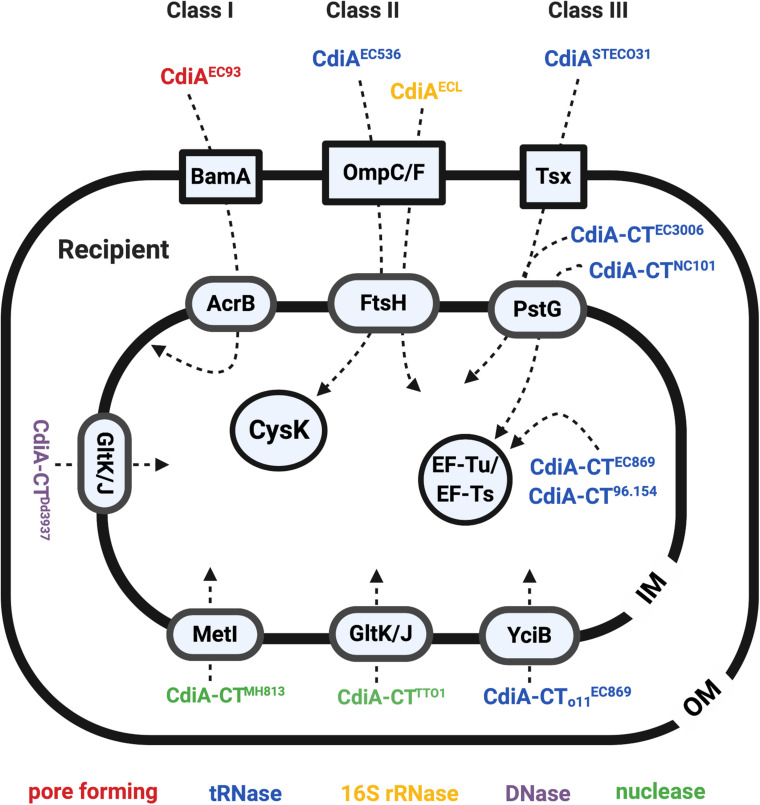
The recipient susceptibility factors that participate in exerting full toxicity of the CdiA. The CdiA toxins were classified into three different classes: the CdiAs that use BamA as outer membrane (OM) receptors are class I effector, the CdiAs that use the OmpC/F are the class II effector, and the ones that use Tsx are the class III effector. The OM, inner membrane (IM), and the cytosol proteins required for full toxicity of the CdiA are labeled in blue boxes in a square, oval, and circle, respectively. The CdiA functions in pore-forming toxins are labeled in red, tRNases are labeled in blue, 16S rRNases are labeled in yellow, DNases are labeled in purple, and the nuclease is labeled in green.

Identification of the binding site between the CdiA^EC93^ toxin and BamA confirmed BamA as the receptor of CdiA^EC93^ (Figure 3). The RBD of CdiA^EC93^ (from Arg1358 to Phe1646) binds to BamA’s loop 6/loop 7 variable region that is identical in hundreds of other *E. coli* strains but shares low-sequence similarity among different CDI-encoding species (Ruhe et al., 2013, 2017). The results correlated well with the observation that CDI^EC93^ is unable to inhibit other CDI homologs-harboring species like *Salmonella enterica* serovar Typhimurium, *Citrobacter freundii*, *Enterobacter aerogenes*, *Enterobacter cloacae*, or *Proteus mirabilis* but was able to inhibit a variety of *E. coli* strains (Aoki et al., 2010; Ruhe et al., 2013). To summarize, the CdiA^EC93^ uses its RBD domain to bind specifically to the OM protein BamA of the *E. coli* recipient, demonstrating that CDI is restricted to intraspecies competition in a recipient receptor-dependent manner.

#### Heterotrimeric OmpC-OmpF but Not BamA Is the Outer Membrane Receptor for CDI^EC536^

In contrast to CDI^EC93^, CdiA of the uropathogenic *E. coli* (UPEC) strain 536 (CdiA^EC536^) was shown to recognize the heterotrimeric OmpC-OmpF complex but not BamA (Figure 3; Beck et al., 2016). More specifically, the RBD region of the CdiA^EC536^ interacts with the extracellular loops L4 and L5 of OmpC (Beck et al., 2016; Ruhe et al., 2017). Unlike the binding region of BamA to CdiA^EC93^, which is highly conserved in protein sequence in hundreds of *E. coli* strains, the L4 and L5 of OmpC is highly diverse in protein sequence even among different *E. coli* strains (Aoki et al., 2008; Beck et al., 2016). Such OmpC polymorphism restricts the range of recipients for CDI^EC536^ (Beck et al., 2016). Although OmpF is strictly required for CDI^EC536^, using *ompF* alleles that are highly diverse from that of EC536 does not interfere with the CDI^EC536^ delivery process, consistent with the hypothesis that the recognition sites reside in OmpC. The data obtained on receptor preference or specificity of CDI from different *E. coli* strains suggest that *E. coli* may use the CDI systems to distinguish “self” from “non-self” cells and promote interactions between siblings.

#### CdiA of CDI^ETECO31^ Binds to the Outer Membrane Receptor Tsx

After identifying the RBD region as the recipient OM receptor binding domain, the RBD region of all identifiable CdiA in the databae resoures of National Center for Biotechnology Information (NCBI) was compared to gain insights in binding specificity and selectivity toward either BamA or OmpC/OmpF (Ruhe et al., 2017). The results indicated that the CdiA RBD region can be divided into four main classes, instead of two, based on their amino acid sequences. Given the variability between the toxin classes, the CdiA^STECO31^ from *E. coli* STEC_O31 was used as a class III effector model to search for its CDI^R^ mutants. The genetic screen of CdiA^STECO31^ CDI^R^ mutants led to the discovery of a new receptor, namely, Tsx (Figure 3), an OM protein that functions as a monomeric nucleoside-specific porin (Bremer et al., 1990). Although the binding region of the Tsx remains elusive, the RBD region of the CdiA^STECO31^ lies in the Gln1385-Tyr1657 (Ruhe et al., 2017).

### Inner Membrane Proteins in Recipient Cells Are Required for Contact-Dependent Inhibition

#### CDI^EC93^ Requires the Inner Membrane Protein AcrB to Exert Its Toxicity

Besides identification of OM receptors, CDI^R^ genetic screens also identified several genes encoding IM components (Figure 3; Aoki et al., 2008; Ruhe et al., 2014, 2017; Willett et al., 2015a). In the CDI^R^ genetic screen using the attacker CDI^EC93^, *acrB* and *bamA* integrity in the prey cells were found mandatory for the attack to be effective. AcrB is an IM multidrug transport protein belonging to the multidrug/proton antiporter that is composed of AcrB, periplasmic protein AcrA, and OM protein TolC (Tikhonova and Zgurskaya, 2004). Intriguingly, only mutations in *acrB* but not *acrA* or *tolC* conferred resistance to the CDI, suggesting that the AcrB-mediated CDI is independent of its multidrug efflux pump function (Aoki et al., 2008). Of note, cells intoxicated with CdiA^EC93^ have reduced proton motive force and steady-state ATP levels, and their AcrB-containing multidrug/proton antiporter function is blocked (Aoki et al., 2009). These results suggested that CdiA-CT^EC93^ might interact with AcrB, thus resulting in dissipation of proton motive force (Aoki et al., 2009). Alternatively, AcrB could anchor the incoming CdiA-CT^EC93^ in the IM to activate the toxin that forms a pore (Jones et al., 2017).

#### CDI^EC536^ and CdiA^ECL^ Require the Inner Membrane Protein FtsH for Toxicity

The recipient’s IM factors required for CdiA^EC536^ is the filamenting temperature-sensitive H (FtsH) protein (Figure 3; Ruhe et al., 2014; Willett et al., 2015a). FtsH is an IM-anchored AAA^+^ protease, and its activity is stimulated by the proton motive force (Akiyama, 2002; Langklotz et al., 2012). As CdiA-CT^EC536^ is a well-defined tRNase that functions in the cytosol (Aoki et al., 2010; Ruhe et al., 2014), the role of FtsH is suggested to mediate toxin translocation across the IM. It is worth noting that CdiA^EC536^ and CdiA^ECL^ both require OmpC-OmpF heterotrimers and FtsH for toxicity (Willett et al., 2015a; Beck et al., 2016). However, the detailed mechanism of how FtsH is involved in CdiA toxicity remains elusive.

#### Inner Membrane Protein PtsG Is Required for Toxicity of CDI^ETECO31^, CDI^NC101^, and CDI^EC3006^

PtsG, the glucose-specific EIICB component of the sugar PTS (sugar phosphoenolpyruvate-dependent phosphotransferase) system, was found to be required for the toxicity of CdiA ^STECO31^ (Gabor et al., 2011). As CdiA-CT^STECO31^ encodes an EndoU anticodon nuclease that claves tRNA^Glu^ in the cytosol (Michalska et al., 2018), the IM protein PtsG is believed to enable CdiA-CT^STECO31^ translocation into the recipient’s cytosol. Further screening for CDI^R^ mutants resisting intoxication for CdiA produced by a variety of different bacterial strains discovered that PtsG is also required for toxicity of CdiA-CT^NC101^ and CdiA-CT^3006^ (Willett et al., 2015a).

#### Multiple Inner Membrane Proteins in Recipient Cells Are Required for Contact-Dependent Inhibition

Additional recipient IM factors were also identified by screening for CDI^R^ mutants resisting intoxication by CdiA produced by a variety of different bacterial strains (Willett et al., 2015a). The screening strategy was designed for identifying entry factors by using chimeric CdiA that harbors the N-terminus from *E. coli* strain EC93 and the C-terminal-containing toxin domain of other strains. The rationale is that the CdiA C-terminus (CdiA-CT) contains a variable domain that specifies the entry pathway into target bacteria and therefore recognizes and exploits specific proteins on the target cell for entry of the CdiA-CT toxin. Such screen has led to the discovery of six “permissive factors” conferring specific entry of different CDI toxins (Table 1 and Figure 3). Besides identifying known IM factor PtsG that is required for CdiA-CT^NC101^ and CdiA-CT^3006^, additional IM proteins including MetI for CdiA-CT^MHI813^, YciB for the orphan CdiA-CT of the EC869 (CdiA-CT_o__11_^EC869^), GltK for *Photorhabdus luminescens* CdiA-CT^TTO1^, and RbsC for *D. dadantii* CdiA-CT^Dd3937^ were uncovered (Figure 3 and Table 1; Willett et al., 2015a). Orphan *cdiA-CTs* encode toxins but have no translation initiation region and therefore are not translated unless grafted with a region encoding an N-terminal CdiA sequence (Poole et al., 2011).

It is worth mentioning that all the identified recipient proteins were IM protein, thus indicating that CdiA-CT is the region recognizing the recipient’s IM receptor but not the OM receptor. This finding is consistent with the evidence that the RBD but not the CdiA-CT region is responsible for binding to the OM receptor of CdiA^EC93^ (Figure 2; Ruhe et al., 2017). The authors also used chimeric CdiA-CT^EC3006–*EC*869o11^ to elucidate which part(s) of the CdiA-CT is responsible for recognition of the cognate IM receptor (Willett et al., 2015a). The CdiA-CT^EC3006–*EC*869o11^ consists of an N-terminal, CdiA-CT^3006^, and C-terminal fragments, CdiA-CT_o__11_^EC869^, and requires PtsG but not YciB for growth inhibition. The results demonstrate that the IM receptor recognition domain of CdiA lies in the N-terminus of CdiA-CT and was thus designated as the entry domain, while the C-terminus of the CdiA-CT is the toxin domain itself (Willett et al., 2015a).

### Recipient Cytoplasmic Factors Recognized by Contact-Dependent Inhibition

#### CDI^EC536^ Requires CysK for Toxicity

Cytoplasmic factors were also found to be required for effective CDI mechanism. In contrast to the roles of OM and IM factors involved in recognition and entry, cytoplasmic factors usually participate in enhancing toxin activity. The cytosolic factor of CdiA^EC536^ is the *O*-acetylserine sulfhydrylase A (CysK) (Figure 3; Diner et al., 2012; Beck et al., 2016). The requirement of CysK in antagonizing recipient growth stems from an unexpected result that CdiA-CT^EC536^ only displays tRNase activity in the presence of CysK both *in vitro* and *in vivo* (Diner et al., 2012). Crystal structure of the CysK/CdiA-CT^EC536^ complex revealed that CysK interacts with the C-terminal Gly-Tyr-Gly-Ile (GYGI) motif of CdiA-CT^EC536^, and this interaction increases the thermostability and tRNase activity of CdiA-CT^EC536^ (Johnson et al., 2016). Intriguingly, CysK also binds to and stabilizes the CdiA-CT^EC536^/CdiI^EC536^ complex in the attacker cell, and such binding reinforces protection against autoinhibition (Kaundal et al., 2016). The CysK/CdiA-CT^EC536^ interaction site mimics the binding site between CysK to its native substrate, CysE. Recent data demonstrated that CdiA-CT^EC536^ has a higher affinity to CysK even in the presence of excess CysE (Johnson et al., 2016; Jones et al., 2017). In brief, CdiA- CT^EC536^ utilizes CysK of the recipient cell to activate its tRNase activity once in the recipient cytosol.

#### Elongation Factor Thermo-Unstable and Elongation Factor Thermo-Stable Are Required for CDI^EC869^, CDI^NC101^, and CDI^96.154^

Other recipient cytosolic factors required for CDI are the Elongation Factor Thermo-Unstable (EF-Tu) and the Elongation Factor Thermo-Stable (EF-Ts) (Figure 3; Jones et al., 2017; Michalska et al., 2017). The CdiA-CT^EC869^ toxin interacts with the EF-Tu/GTP/tRNA complex with high affinity. More importantly, the tRNase activity of CdiA-CT^EC869^ was only observed in the presence of this complex under *in vitro* conditions (Jones et al., 2017). Although EF-Ts was dispensable in activating CdiA-CT^EC869^ *in vitro*, it is required *in vivo*. The role of EF-Ts *in vivo* was proposed to be promoting the formation of the EF-Tu/GTP/tRNA complex. Aside from EC869, CdiA-CTs from strains NC101 and 96.154 also interact with EF-Tu, and both were unable to intoxicate a *tsf* mutant that lacks EF-Ts (Jones et al., 2017; Michalska et al., 2017). It is worth noting that CdiA-CT^EC869^, CdiA-CT^NC101^, and CdiA-CT^96.154^ share low-sequence similarity, suggesting that hijacking EF-Tu for activation may be a common strategy used by CDI toxins (Jones et al., 2017; Michalska et al., 2017).

As summarized above, the CDI system requires recipient membrane receptors and cytosolic activators to exert full toxicity. Exemplified by *E. coli* strains, a wide variety of the RS factors participates in recognition (OM receptor), translocation (IM proteins), and activity (cytoplasmic factors) of CDI toxins. Many other organisms harbor functional CDI, and they differ in gene organization, protein sequence, and cytotoxicity (Aoki et al., 2010; Kiel et al., 2012; De Gregorio et al., 2018; Allen and Hauser, 2019). As such, it is anticipated that novel CDI-dependent recipient receptors and activators would likely be discovered in future studies.

## Type VI Secretion System

### The Discovery, the Players, and the Type VI Secretion System “Firing” and Mode of Action

The T6SS was initially coined to be a virulence factor targeting eukaryotic hosts in many Gram-negative bacteria (Figure 1; Mougous et al., 2006; Pukatzki et al., 2006, 2007; Schell et al., 2007). Subsequent studies revealed that the T6SS could also target prokaryotic cells (Hood et al., 2010; MacIntyre et al., 2010; Schwarz et al., 2010). One of the first demonstrations came from *P. aeruginosa* on one of its T6SS substrate/effector Tse2 (Hood et al., 2010). Tse2 was toxic to *E. coli* and *Burkholderia thailandensis* when expressed ectopically, and this toxicity can be neutralized by the gene product encoded immediately downstream of *tse2*. The downstream gene was therefore named the *tse2 immunity* (*tsi2*). The authors also demonstrated that a *P. aeruginosa* strain lacking *tse2-tsi2* lost fitness against its parental strain when the two strains were cocultured on solid but not liquid media and that this could be complemented by providing a plasmid-borne *tsi2*. The results demonstrated that T6SS uses the Tse2 toxin to gain fitness against a Tsi2-lacking sibling, and this occurred in a contact-dependent manner. In *Vibrio cholerae*, the T6SS-dependent antibacterial activity was shown against many Gram-negative bacteria, including *Salmonella typhimurium*, *Citrobacter rodentium*, and *E. coli* (MacIntyre et al., 2010). It has also been demonstrated that T6SS toxins can intoxicate a wide range of organisms including bacteria, archaea, fungi, and eukaryotic hosts (Coulthurst, 2019; Klein et al., 2020).

**FIGURE 4:**
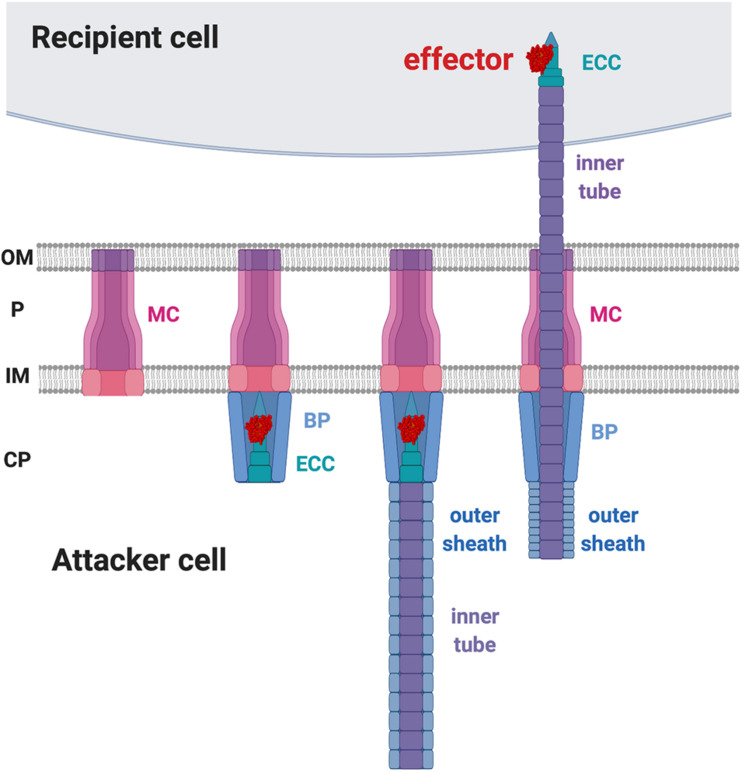
Type VI secretion system (T6SS) working model. The first step of T6SS assembly is the formation of the membrane complex (MC). The second step is the recruitment of the baseplate (BP) complex and the effector-containing complex (ECC) to the MC. The third step is the polymerization of the inner tube and the outer sheath inside the attacker cytosol. Upon trigger, the outer sheath contracts and propels the inner tube to penetrate the recipient membrane. OM, outer membrane; P, periplasm; IM, inner membrane; CP, cytoplasm.

In contrast to CDI that employs a receptor-based recognition mechanism for toxin delivery at intraspecies levels, T6SS appears not to depend on a specific receptor for toxin delivery. T6SS’s action mold could explain it’s ability to target multiple organisms. The T6SS is composed of 13–14 core Type six secretion (Tss) proteins that are assembled in a structure highly similar to a contractile phage tail (Chang et al., 2017; Rapisarda et al., 2019; Wang et al., 2019). The current T6SS working model suggests that the formation of the membrane complex (MC) across the inner and outer membranes of the attacker cell is the first step in the assembly process (Figure 4). The membrane complex composed of (TssJ-)TssL-TssM (Ma et al., 2009; Rapisarda et al., 2019) functions as a scaffold for the recruitment of the baseplate (BP) complex and the effector-containing complex (ECC) for the initiation of the T6SS assembly (Brunet et al., 2015; Wang et al., 2019). The structure of the ECC resembles the tip of a spear that can puncture recipient cells (Basler and Mekalanos, 2012; Brunet et al., 2013). The BP serves as the docking site of the ECC and guides it to the MC. The BP is composed of TssE-TssF-TssG-TssK, and the ECC is composed of VgrG-(PAAR)-(adaptor)-effectors (Felisberto-Rodrigues et al., 2011; Brunet et al., 2015). The loading of the spear tip complex is believed to trigger polymerization of the spear handle that is composed of the Hcp inner tube and the TssB-TssC outer sheath (Figure 4; Mougous et al., 2006; Basler et al., 2012; Lossi et al., 2013; Zhang et al., 2013; Liang et al., 2019; Wu et al., 2020). When triggered, the outer sheath contracts and propels the inner tube and the “spear tip,” ECC, likely through the membrane complex scaffold to puncture the membrane of a recipient cell (Basler et al., 2012, 2013). The collective knowledge suggests that the toxin delivery to the recipient cell is through a mechanical force, rather than upon specific receptor binding (Figure 4).

### Recipient Signals Triggering the Type VI Secretion System

#### Type VI Secretion System “Fires” in Response to Membrane Assaults

**FIGURE 5:**
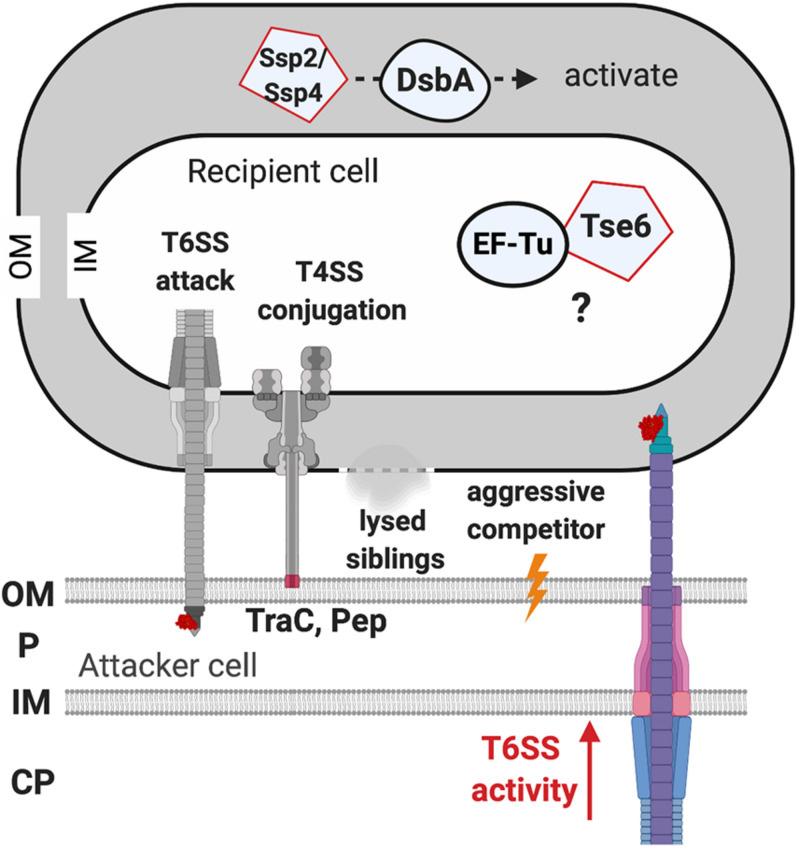
The recipient susceptibility factors enhancing the outcome of the type VI secretion system (T6SS) attack. The T6SS attack from the recipient cell triggers a T6SS counterattack. The T4SS-mediated membrane disruption that is caused by the adhesin TraC and Pep is required for activating the T6SS attack. Lysed siblings, aggressive competitors, and the non-self-competitors also trigger a T6SS attack. The elongation factor thermo-unstable (EF-Tu) of the recipient cell may be required for Tse6 to exert full toxicity, but the mechanism remains elusive. The DsbA of the recipient cell is required to activate the Ssp2 and Ssp4 periplasmic toxins. OM, outer membrane; P, periplasm; IM, inner membrane; CP, cytoplasm.

The initial clues for recipient factors affecting the outcome of T6SS killing came from microscopic observations of T6SS firing events (Basler and Mekalanos, 2012; LeRoux et al., 2012). T6SS firing events were monitored by visualization of ClpV-GFP as ClpV is required for disassembly of contracted T6SS sheath, an event subsequent to T6SS firing (Bönemann et al., 2009; Pietrosiuk et al., 2011). The presence of ClpV-GFP foci thus indicates that T6SS firing has just happened (Mougous et al., 2006; Basler and Mekalanos, 2012). It was observed that *P. aeruginosa* ClpV-GFP foci occurred at the exact place where its neighboring sibling cells also had a ClpV-GFP foci, indicating that one of the activating signals for *P. aeruginosa* T6SS firing is the T6SS attack from a neighbor sibling cell (Basler and Mekalanos, 2012; LeRoux et al., 2012). This phenomenon was then demonstrated in an interspecies T6SS competition scenario. When punctured by the *V. cholerae* T6SS, *P. aeruginosa* fires back using its T6SS at the exact position where it was challenged (Basler et al., 2013). Similarly, *Agrobacterium tumefaciens* T6SS also triggers a *P. aeruginosa* counterattack, which led to higher killing of T6SS-active *A. tumefaciens* as compared to T6SS-inactive strains (Ma et al., 2014). Interestingly, the T6SS counterattack also occurs when sensing the pKM101 T4SS mating pair formation (Mpf) system of *E. coli* donor cells to resist T4SS-mediated gene transfer of foreign DNA (Ho et al., 2013). Because T6SS firing is also induced by membrane-disrupting compounds such as polymyxin B, the authors concluded that the T6SS counterattack results from Mpf-mediated membrane disruption. Recent studies further showed that the production of two adhesins (TraC and Pep), or the formation of a T4SS channel, but not assembly of conjugative pilus, is capable of activating a T6SS counterattack (Gordon et al., 2017; González-Rivera et al., 2019). Therefore, T6SS firing could be a defensive weapon in response to various assaults challenging membrane integrity (Figure 5).

#### Kin Discrimination Regulating the Type VI Secretion System Attack

It has been demonstrated in multiple systems that the T6SS attack could be fine-tuned in response to different recipient cells (Ma et al., 2014; LeRoux et al., 2015; Lazzaro et al., 2017; Wu et al., 2019). For example, in *P. aeruginosa*, a non-self-recipient cell triggers a stronger T6SS attack than a susceptible sibling (LeRoux et al., 2015). Furthermore, the *P. aeruginosa* T6SS activity monitored by ClpV1-GFP was significantly elevated when cocultured with *B. thailandensis* as compared to a monoculture (LeRoux et al., 2012). The authors demonstrated that *P. aeruginosa* senses a “danger signal” released by lysed sibling cells and activates its T6SS to launch a counterattack (LeRoux et al., 2015). The enhanced T6SS susceptibility triggered by non-self-recipient cells was also demonstrated in *A. tumefaciens* (Ma et al., 2014). *A. tumefaciens* only exhibits antibacterial activity against *E. coli* but not against susceptible siblings *in vitro* (Ma et al., 2014). Furthermore, *A. tumefaciens* tends to antagonize other competitive *A. tumefaciens* strains from different genomospecies but not to the same degree to those within the same genomospecies *in planta* (Wu et al., 2019). In *Serratia marcescens*, the transcription level of T6SS is fine-tuned as the T6SS transcript level of *S. marcescens* varies when challenged by different competitors. Only basal levels of T6SS transcripts were detected when confronted with harmless recipient cells, while upregulation occurs at moderate or higher levels when confronted with contender or aggressive competitors (Lazzaro et al., 2017). Overall, these findings unveil the importance of kin recognition in determining the outcome of the T6SS attack, but future systematic analysis is required to identify the genetic features or determinants governing the fate of a competition (Figure 5).

### Recipient Cell Factors Involved in Type VI Secretion System Toxicity

#### Elongation Factor Thermo-Unstable Could Be the Recipient Susceptibility Factor for *Pseudomonas aeruginosa* Tse6

The first evidence for the involvement of specific T6SS RS factors came from the characterization of the *P. aeruginosa* effector Tse6-loaded complex, which consists of Tse6, Tsi6 immunity protein, VgrG1, effector-associated gene with *tse6* (EagT6), and EF-Tu (Whitney et al., 2015). The presence of EF-Tu in the Tse6-loaded complex was unexpected, and the authors addressed the role of EF-Tu by proposing four possibilities: EF-Tu may be required for (1) stabilizing Tse6, (2) activating Tse6, (3) facilitating Tse6 export from attacker cell, or (4) entering recipient cell. After ruling out the first three, the authors deduced that the interaction of Tse6 with EF-Tu might be required for entering the recipient cell. However, further study on the ability of the Tse6-loaded complex to translocate across membranes using liposome-based *in vitro* translocation assay showed that Tse6 translocation happened spontaneously in the absence of the inner-face EF-Tu (Quentin et al., 2018). Thus, EF-Tu may not play a role in entering recipient cells across the lipid bilayer, and the exact role of EF-Tu in the interbacterial competition is still to be elucidated.

#### DsbA Is Required for Effectiveness of the Type VI Secretion System Effectors Ssp2 and Ssp4 From *Serratia marcescens*

Another example of RS factors affecting T6SS toxicity is DsbA that functions as a periplasmic disulfide bond-forming protein (Mariano et al., 2018). *S. marcescens* has two DsbAs, DsbA1 and DsbA2, which are functionally redundant for a proper T6SS functionality. Indeed, in *S. marcescens*-secreting cells, the presence of either DsbA1 or DsbA2 is sufficient for T6SS activity, but T6SS assembly and secretion levels are significantly compromised in the absence of DsbA1 and DsbA2. Strikingly, the peptidoglycan hydrolase Ssp2 and Ssp4 (English et al., 2012) are able to inhibit Ssp2- and Ssp4-susceptible *S. marcescens* strains, while a recipient lacking both DsbA1 and DsbA2 was entirely resistant against the activity of these periplasmic-acting effectors (Mariano et al., 2018). The requirement of DsbA for the toxicity of Ssp2 and Ssp4 was also confirmed by artificially expressing and targeting Ssp2 and Ssp4 to the *E. coli* periplasm, in which their toxicity is relieved if the *E. coli* strain lacks *dsbA*. Attacker cells expressing disulfide bond-lacking Ssp2 or Ssp4 did not show T6SS-mediated antibacterial activity. It is generally believed that T6SS delivers effectors from the attacker cell’s cytoplasm directly into the recipient cell, Ssp2 and Ssp4 effectors are unlikely to localize in the attacker cell’s periplasm to form a disulfide bond before its delivery. Thus, it remains unknown how DsbA influences T6SS activity in the attacker cell, but the contribution of DsbA or disulfide bond formation for activity of incoming periplasmic toxins in the recipient cell is likely a widespread mechanism (Figure 5; Mariano et al., 2018).

#### Identification of the Recipient’s ClpAP in Enhancing *A. tumefaciens* Type VI Secretion System Killing

In *A. tumefaciens*, a high-throughput screening (HTS) aiming to identify RS factors that affect the T6SS killing outcome was performed (Lin et al., 2020). Using *E. coli* K12 strain BW25113 as the model recipient cell, several RS factors that enhance *E. coli* susceptibility to *A. tumefaciens* T6SS attack were identified. To date, the confirmed RS-encoding genes include *clpA*, *clpP*, *gltA*, *ydhS*, *ydaE*, and *cbpA*, all encoding cytosolic proteins. These results suggest that the RS factors affecting *A. tumefaciens* T6SS killing outcome are rather involved after injection of T6SS toxins into the recipient cells.

The *clpP* gene encoding ClpP protease is universal and highly conserved in both prokaryotes and eukaryotic organelles. Its activity depends on other adapter proteins such as ClpA or ClpX AAA^+^ ATPase for substrate recognition (Bhandari et al., 2018; Figaj et al., 2019). The authors showed that *clpA* but not *clpX* is required for enhancing susceptibility to *A. tumefaciens* T6SS killing, suggesting the involvement of ClpAP. ClpP variants deficient in ClpP protease activity or incapable of interacting with its adaptor protein could not restore T6SS effectiveness against a *clpP* knockout mutant, suggesting that ClpA–ClpP interaction and subsequent proteolysis are critical in enhancing susceptibility to T6SS killing. While the mode of action of recipient ClpAP complex involved in enhancing T6SS killing remains unknown, three hypotheses could be proposed for further testing. First, ClpAP complex may be used to enhance toxin activity, such as the Tde1 and/or Tde2 DNase activity, the major T6SS antibacterial weapons of *A. tumefaciens* strain C58 (Ma et al., 2014) used for the screen. Second, ClpAP complex could be hijacked by *A. tumefaciens* to trap or degrade an *E. coli* defense protein from inhibiting the activity of an incoming toxin. The third hypothesis is that the absence of a ClpAP system may result in substrate accumulation that interferes with T6SS firing or toxin activity of the attacker.

In summary, based on the broad spectrum of recipient cells that T6SS toxins act on, T6SS appears not to require recipient receptor for protein toxin entry. Current evidence suggests that specific RS factors may rather be used for the full activation of T6SS toxins once entering the recipient cells (Figure 5). However, future studies on the mode of action of identified RS factors and more comprehensive genetic screens are required to answer these questions. Besides RS factors, recent studies have revealed the presence of immunity-independent resistance in recipient cell that were nicely reviewed by Robitaille et al. (2020). These recipient defense factors or mechanisms include physical barriers such as exopolysaccharide (Toska et al., 2018), envelope stress responses (Hersch et al., 2020), or peptidoglycan editing (Le et al., 2020). Growing evidence of the involvement of recipient factors in either enhancing T6SS toxicity or defense against T6SS indicates an evolutionary arms race during interbacterial competition, which may play roles in shaping microbiome.

## New Player: Type I Secretion System Cdz

Recently, a novel Cdz system that requires the canonical T1SS proteins CdzA and CdzB has been described in *Caulobacter crescentus* (García-Bayona et al., 2017). The *cdz* operon consists of five genes encoding two T1SS components (CdzA IM component and CdzB membrane fusion protein) followed by two-peptide toxin (CdzC and CdzD) and the immunity protein CdzI (García-Bayona et al., 2017, 2019). The CdzC/CdzD two-peptide toxin kills the target cell by membrane depolarization, and its toxicity is neutralized by the immunity protein CdzI (García-Bayona et al., 2017). In contrast to log phase-specific CDI, the Cdz is stationary phase-specific. The transcript of the *cdz* operon and the gene products are highly induced in the stationary phase, while the Cdz protein levels are not detectable in the log phase.

The Cdz system is not species-dependent and can antagonize other closely related species. The Cdz of *C. crescentus* was able to inhibit a CdzI immunity protein-lacking sibling, *Caulobacter segnis*, and *Brevundimonas subvibrioides* sp. Poindexter. However, the *C. crescentus* Cdz was not able to antagonize *Asticcacaulis excentricus*, which also belongs to the *Caulobacteraceae* family but is more distantly related to *C. crescentus*, and other even more distantly related bacteria like *A. tumefaciens* or *E. coli* (García-Bayona et al., 2017). This implied that the T1SS-mediated growth inhibition by Cdz only occurs between close-related lineage but at broader scope than CDI. As the Cdz system can be found in Firmicutes, alpha-proteobacteria, beta-proteobacteria, and particularly widespread in gamma-proteobacteria, the Cdz is another common contact-dependent antibacterial strategy used by bacteria to thrive in the environment (García-Bayona et al., 2017).

A more recent study searching for recipient cells resistant to *C. crescentus* Cdz-killing led to the identification of a previously uncharacterized gene *ccna_01968* (García-Bayona et al., 2019). The *ccna_01968* was renamed as the *pentapeptide envelope resistance A* (*perA*) gene as it encodes a quadrilateral beta-helix protein. Biochemical data and microscopy observation demonstrated that PerA is a surface-exposed OM protein. The *perA* mutant strains resistant to Cdz were sensitized again by expressing *perA in trans*, suggesting that PerA may act as the receptor of CdzC/CdzD toxin (García-Bayona et al., 2019). The mode of action of PerA and whether additional RS factors in recipient cells are involved in Cdz-mediated antibacterial activity require further in-depth molecular studies and genetic screens.

## Strategies for Identifying Recipient Factors

The approaches used to identify RS factors were mostly by screening mutant libraries for resistant recipient cells. This led to the discovery of multiple RS genes involved in maximizing the toxicity of CDI, T6SS, or T1SS Cdz. Other RSs were identified through knowledge-based approaches such as on the basis of their association with the toxin either physically or biochemically. Here, we summarize the methods used and discuss potential strategies for the discovery of novel recipient factors.

### Phenotype-Based Method: Mutant Library Screening

Genetic screen is proven to be a powerful and non-biased method for identifying RS factors, which is applicable to any contact-dependent antibacterial system. As summarized in Table 2, the selection of resistant strains can be screened from identifying the survivors of a mutant library pool cocultured with attacker/recipient cells. The mutations responsible for the resistance phenotype can be later identified by complementation using a genomic library (Aoki et al., 2008; Ruhe et al., 2014, 2017; Willett et al., 2015a; Jones et al., 2017) or by whole-genome sequencing (García-Bayona et al., 2019). With the availability of the *E. coli* Keio library containing 3,909 knockout mutant strains (Baba et al., 2006), an HTS with the aid of pipetting robot and 96-well systems was established to screen *E. coli* recipient factors (Lin et al., 2020). Such screen can lead to the immediate identification of gene of interest without complementation by a genomic library and/or sequencing. However, the use of knockout mutant library cannot identify RS genes that are essential for bacterial growth. Thus, CRISPR interfering (CRISPRi) using a catalytic null mutant of the Cas9 endonuclease, dCas9, and guide RNA (gRNA) library (Cui et al., 2018) serves as an alternative and complementary method to screen for recipient factors that are not available in knockout or Tn-insertion mutant libraries. The availability of *E. coli* CRISPRi gRNA library (Addgene, Watertown, MA, United States) created by the Bikard lab enables such screen in *E. coli* and can be expanded to other bacterial species. A series of broad host range vectors that carry the d*cas9* gene under control of the *ptet* promoter and the gRNA under control of a constitutive promoter are available for future applications in many Proteobacterial species (Depardieu and Bikard, 2020).

**TABLE 2:** Summary of current and potential methods for discovery of recipient susceptibility (RS) factors.

Approach	Method	Pros	Cons	Secretion system: recipient factors	References
Phenotype-based method	Screen resistant strains from a mutant library pool	Fast and robust; phenotype-dependent	A selectable phenotype is required	CDI: BamA, OmpC-OmpF, Tsx, AcrB, FtsH, PtsG, MetI, YciB, GltK, and RbsC Cdz: PerA	Aoki et al., 2008; Willett et al., 2015a; Beck et al., 2016; Ruhe et al., 2017; García-Bayona et al., 2019
	Screen resistant strains of individual mutants from a mutant library	linking the gene to phenotype directly	An HTS platform is required to reduce the labor and time	T6SS: ClpA and ClpP	Lin et al., 2020
Knowledge-based method	Identify toxin-interaction proteins *via* protein– protein interaction assays: 1. Co-purification 2. BTH 3. YTH	Direct and fast in detecting physical interactions	Antibody or detection tools for proteins of interest are required; identified proteins may not function as a RS factor	CDI: EF-Tu and CysK T6SS: EF-Tu	Diner et al., 2012; Whitney et al., 2015; Jones et al., 2017; Quentin et al., 2018
	Identify proteins activating toxin activity	Direct and fast, without large-scale analysis or screening	Prior knowledge or hypothesis is required	T6SS: DsbA	Mariano et al., 2018

*CDI, contact-dependent inhibition; EF-Tu, elongation factor thermo-unstable; HTS, high-throughput screening; OM, outer membrane; T6SS, type VI secretion system.*

### Knowledge-Based Method

#### Identification via Protein–Protein Interaction

The major roles of RS factors are in recognition, entry, or activation of the toxins. Thus, an approach to identify recipient factors is to search for toxin-interacting proteins. Indeed, EF-Tu, the common RS factor involved in CDI and potentially for T6SS, was identified as one of the components taking part in the toxin–immunity protein complexes (Jones et al., 2017). Thus, co-expression of toxin–immunity complex followed by co-immunoprecipitation or pulldown assay (Brymora et al., 2004; Kaboord and Perr, 2008; Lin and Lai, 2017a) can lead to the discovery of toxin-interacting proteins. This serves as a straightforward method to identify RS factors that may play a role in toxin entry or activation. In addition, the toxin proteins can be used as a bait in well-established protein–protein interaction platforms such as bacterial two-hybrid (BTH) (Battesti and Bouveret, 2012) or yeast two-hybrid (YTH) (Mehla et al., 2015; Lin and Lai, 2017b) to identify potential RS factors by screening a recipient genomic library.

#### Identification via Activating Toxin Activity

RS factors that are hijacked to activate toxin activity can be identified based on the knowledge of the toxin’s mode of action. For example, periplasmic disulfide bond-forming protein DsbA that is known to be required for folding or stabilization of proteins located in the periplasm could be critical for activity of periplasmic bacterial toxins such as peptidoglycan hydrolases and phospholipase (Kadokura and Beckwith, 2010). Based on this knowledge, the role of DsbA in T6SS-mediated antibacterial activity of *S. marcescens* was investigated and found to be required for the activity of the peptidoglycan hydrolase Ssp2 and the periplasmic toxin Ssp4 (Mariano et al., 2018). It is possible that DsbA plays a broader role for toxin activation delivered by multiple antibacterial systems. Besides DsbA, involvement of the ClpAP protease in T6SS susceptibility (Lin et al., 2020) also suggested that various types of proteases may be used for activating toxin activity by either cleaving full-length toxin proteins into more active truncated forms or degrading proteins that may inhibit toxin activity. A recent report showed that self-cleavage at both the N- and C- termini of an Rhs-family T6SS toxin *Tse*I is not required for secretion but critical for its toxin activity (Pei et al., 2020). This finding also suggests that protease cleavage could be a strategy used for toxin activation in the recipient cell. Since the mechanism for N-terminal cleavage of *Tse*I remains unknown, it may be mediated by an unknown protease residing in the recipient cell. Future work to test these potential modifying enzymes in activating antibacterial toxins of various systems shall shed light to understand the molecular basis of toxin action once they are translocated into the recipient cells.

## Discussion

Understanding the mode of action of antibacterial toxins and their target spectra may help us develop novel antibacterial therapies in biomedical and agricultural applications (Sana et al., 2017; Bernal et al., 2018; Trunk et al., 2018; Khakhum et al., 2019; Allsopp et al., 2020). For example, accumulating evidence indicated that T6SSs in commensal bacteria such as *Bacteroides fragilis* and *Pseudomonas protegens* play a critical role in the defense against invading bacterial pathogens and impact microbial community in the gut of mammalian and insect, respectively (Chatzidaki-Livanis et al., 2016; Wexler et al., 2016; Vacheron et al., 2019). T6SS is also widespread in plant-associated beneficial bacteria such as *Pseudomonas putida* and *Pseudomonas fluorescens* functioning as a biocontrol agent in protecting plants with their antagonistic activity against bacterial and fungal pathogens (Decoin et al., 2014; Bernal et al., 2017). However, these beneficial bacteria are also susceptible to killing by competitor bacteria equipped with antibacterial weapons. Thus, engineering commensal bacteria to protect from or defend against pathogenic bacteria in a polymicrobial community may be beneficial for human and plant health.

**FIGURE 6:**
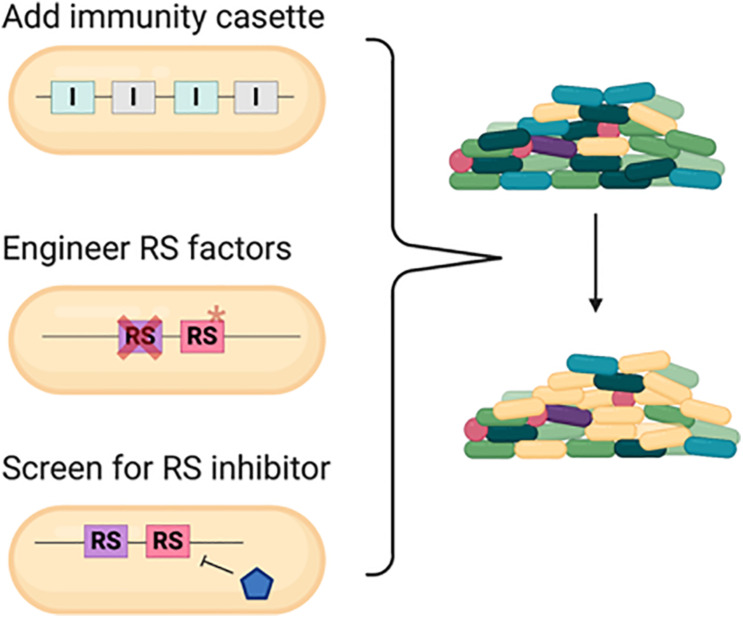
Strategies to engineer commensal bacteria for protection from or defense against pathogens in a polymicrobial community. Conventional strategy is to add an array of immunity gene cassette to the commensal bacteria. With the understanding of the recipient susceptibility (RS) factor, disrupting the RS factor and/or screening for specific inhibitor to conditionally inhibit the RS factor could also serve as novel methods. Cross represents deletion and asterisk represents mutation.

Based on the current knowledge, we proposed three strategies for defense against pathogens in a polymicrobial community (Figure 6). One conventional way is engineering strains with specific or arrays of various immunity genes that may offer broad-spectrum protection (Sana et al., 2017; Trunk et al., 2018; Khakhum et al., 2019). With the understanding of the RS factors, alternative approaches could be designed in these commensal bacteria with better survival and competitive capacity. First, engineering the strains with deletion or point mutation in the common RS gene can increase the resistance against killing from various bacteria harboring multiple antibacterial weapons. The common RS factor EF-Tu utilized by both CDI and perhaps T6SS for enhanced killing is a potential RS target. However, since EF-Tu is an essential gene, the detailed molecular mechanisms and amino acid residues critical for toxicity enhancement are required prior to engineer the EF-Tu variant combining proper physiological function and resistance to antibacterial killing. Second, these RS factors can be ideal targets to screen natural products or synthetic chemicals to shut down their expression or ability in enhancing toxin entry or activity. This method offers advantages to bypass genetic modification and more flexibility in temporal and spatial control for such applications.

## Concluding Remarks

In conclusion, bacteria have deployed versatile bacterial secretion systems as antibacterial weapons for fitness and survival. Similar to the arms race between hosts and pathogens, the bacterial attackers evolve to recognize or hijack recipient cell factors to maximize the antagonism by enhancing the entry or toxicity of bacterial toxins. It is also worth mentioning that some of the recipient proteins are attacking “hotspots.” For example, the OmpC/OmpF OM receptor is the target of both CdiA^EC536^ and CdiA^ECL^, the IM receptor PstG is the common translocator for multiple CdiA proteins, and the cytoplasmic factor EF-Tu is targeted by multiple CdiA proteins and perhaps Tse6 (Table 1). For receptor-mediated recognition and antibacterial activity at intraspecies levels, different secretion systems tend to target the same or highly similar receptors. We argue that future identification of more RS factors involved in toxins transported by different secretion systems may reveal more toxins targeting “hotspots” to further accelerate the development of novel antibacterial therapies in biomedical and agricultural applications.

## Author Contributions

H-HL, AF, and E-ML conceived the review. H-HL and E-ML wrote the first draft. All the authors contributed to complete the final version of the manuscript.

## Conflict of Interest

The authors declare that the research was conducted in the absence of any commercial or financial relationships that could be construed as a potential conflict of interest.
